# The Relative Contributions of Different Chemical Components to the Oxidative Potential of Ambient Fine Particles in Nanjing Area

**DOI:** 10.3390/ijerph18062789

**Published:** 2021-03-10

**Authors:** Xiaoyun Ma, Dongyang Nie, Mindong Chen, Pengxiang Ge, Zhengjiang Liu, Xinlei Ge, Zhirao Li, Rui Gu

**Affiliations:** 1 Collaborative Innovation Center of Atmospheric Environment and Equipment Technology, Jiangsu Key Laboratory of Atmospheric Environment Monitoring and Pollution Control, School of Environmental Science and Engineering, Nanjing University of Information Science & Technology, Nanjing 210044, China; xiaoyunma@nuist.edu.cn (X.M.); 20181114077@nuist.edu.cn (P.G.); 20191212016@nuist.edu.cn (Z.L.); 002565@nuist.edu.cn (X.G.); 20181212010@nuist.edu.cn (Z.L.); 2 School of Atmospheric Sciences, Nanjing University, Nanjing 210023, China; dynie@nju.edu.cn; 3 Siegwerk Shanghai Ltd., Shanghai 201108, China; ruigu710@163.com

**Keywords:** ambient fine particles, oxidative potential, dithiothreitol, excitation-emission matrix

## Abstract

Ambient fine particles (PM_2.5_) have been shown to have adverse health effects by inducing oxidative stress. Here, dithiothreitol (DTT)-based oxidative potential (OP) was used to assess the capacity of oxidative stress caused by PM_2.5_. In this study, PM_2.5_ samples were collected in the Nanjing area in 2016, and physicochemical properties and DTT activity were investigated. The annual mean PM_2.5_ mass concentration was 73 μg m^−3^ and greatly varied among seasons (spring > winter > summer > autumn). Three fluorescent substances were identified by the excitation-emission matrix (EEM) spectrum. The annual mean mass-normalized DTT activity (DTT_m_; 0.02 nmol min^−1^ μg^−1^) was similar to that documented for cities of some developed countries. The annual mean volume-normalized DTT activity (DTT_v_) showed a relatively high value of 1.16 nmol min^−1^ m^−3^, and the seasonal mean DTT_v_ was highest in winter, followed by spring, autumn, and summer, whose pattern is different from PM_2.5_ mass concentration. Correlation and multiple linear regression analysis suggested that transition metals may have a greater effect on OP in autumn and winter, humic-like substances and UV absorbing aromatic substances may have a strong effect on OP in spring and summer. Generally, this study enhances our understanding of seasonal variation in health effects associated with PM_2.5_.

## 1. Introduction

Atmospheric fine particles (diameter ≤ 2.5 μm, PM_2.5_) have affected air quality and human health in China for years [1]. Recent studies have found that disease such as cardiovascular disease [2,3], respiratory disease [4], mental stress, and premature death are associated with atmospheric fine particulate matter [5]. Exposure to ambient particulate matter induces the production of reactive oxygen species (ROS) in biological systems, including superoxide anions (·O_2_^−^), hydroperoxides (·HO_2_), and hydroxyl radicals (·OH), and these ROS are the main cause of the adverse health effects. Excess ROS disrupts the redox state, causing oxidative stress (OS) and leading to cell apoptosis, biological senescence, and various diseases [6,7]. The ability of particulate matter to induce the production of ROS in organisms is called oxidative potential (OP). Methods of assessing the OP of particulate matter are divided into two categories: cellular and acellular methods. Acellular methods for redox activity are widely used because they are convenient and cost-effective and include the dithiothreitol (DTT), ascorbic acid (AA), and glutathione (GSH), as well as electron spin resonance (ESR) assays [8]. ESR measures the generation of hydroxyl radicals via electron spin resonance, whereas the DTT, AA, and GSH assays measure the depletion rate of chemical proxies for cellular reductants (DTT) or antioxidants (AA, GSH), which is proportional to the generation rate of ROS. Particle-bound ROS measurements use fluorescent-based techniques to measure concentrations of specific ROS, usually the hydroxyl radical or hydrogen peroxide, on or within a PM sample. The development of particle-bound ROS measurements drives the development of acellular OP assays, but recent studies have demonstrated the relevance of OP to health. In Canada, PM_2.5_ oxidative burden was shown to be more closely associated with lung cancer mortality than PM_2.5_ mass concentration using the GSH assay [9]. In Krakow, average values of OP were higher for the PM_2.5_ fraction than for the PM_10_ fraction based on the AA assay [10]. Quantification of the OP of PM_2.5_ by hydroxyl radicals in Beijing and Wangdu revealed that OP was higher on polluted days and higher in Wangdu (a suburb) than in Beijing [11].

The DTT assay is the most efficient and rapid method for assessing the OP of particulate matter. DTT is a strong reducing nucleophilic reagent that can replace intracellular thiol antioxidants [12,13]; the chemical components of PM can catalyze electronic transport from DTT to oxygen, generating ·O_2_^−^ radical. Particulate OP can be evaluated by determining DTT consumption rates [2,14,15]. In the U.S., DTT activity was found to be higher in winter and was correlated with organic and metal species [16]. The OP of PM_2.5_ was measured by the DTT method in Wuhan and was found to be significantly higher than the OP of PM_2.5_ in developed countries. The major sources of OP variation in China were shown to be associated with sources such as the combustions of different fuels and dust emissions [17].

In recent years, some transition metals such as Cu, Mn, V, and Ni, as well as some organics such as quinones and polycyclic aromatic hydrocarbons (PAHs), in ambient particles have been found to undergo redox reactions with DTT [14,18,19]. Humic-like substances (HULIS) have been shown to be important redox-active substances that act as electron carriers for ROS generation. HULIS have high molecular weights, complex chemical structures, and high DTT reactivity because of their higher polarity [20,21]. Therefore, the investigation of chemical component contribution to OP will be a relevant indicator to identify the high potential toxic effects on human health.

Research on the OP of particulate matter in the Yangtze River Delta region (YRD), especially in Nanjing (a major city in the YRD) is still lacking. There is thus a need to study the air quality and associated health risks in Nanjing. Here, the OP of PM_2.5_ was measured in Nanjing from March to December 2016, and multiple linear regression (MLR) was used to quantify the contributions of the PM_2.5_ components to OP. Correlations of single and composite components with the OP were also examined. The focus of this study was on water-soluble DTT activity, which is linked to the adverse health impacts of PM_2.5_ [22]. Water-soluble ROS can be readily absorbed and transported by the body because of its solubility and is associated with acute oxidative stress [23]. Furthermore, the chemical composition of PM_2.5_ was measured, temporal variation in the water-soluble OP of PM_2.5_ was explored, and the contribution of each component of PM_2.5_ to the OP was evaluated. Generally, this study provided basic data that enhanced our understanding of the health risks associated with PM_2.5_ in Nanjing [24].

## 2. Materials and Methods

### 2.1. PM_2.5_ Mass Concentration and Chemical Components

A total of 131 daily PM_2.5_ quartz filter (1851-865, Whatman^TM^, GE Healthcare Life Sciences, Maidstone, UK) samples (spring: *n* = 49, summer: *n* = 30, autumn: *n* = 22, winter: *n* = 30) were collected at Nanjing University of Information Science and Technology (32.20° N, 118.71° E). This site is located in the northern suburbs of Nanjing and is surrounded by residential areas, several chemical plants, and a two-way six-lane highway approximately 1200 m away from the east side. A high flow sampler (TE-5028A, Tisch Environmental, Cleves, OH, USA) with an air flow rate of 1.13 m^3^ min^−1^ was used. Sampling was conducted continuously (i.e., 24 h a day) from 15 March 2016 to 28 December 2016. The quartz filters were thermally treated at 450 °C for 4 h in advance to remove organic matter and were weighed at constant temperature and humidity before and after sampling. After sampling, filters were wrapped in tin foil, stored at −20 °C, and protected from light until analysis.

### 2.2. Sample Preparation

A total of 12.56 cm^2^ of each filter was punched and extracted by sonication (KQ2200DE, Kunshan Ultrasonic Instruments, Kunshan, China) with 40 mL of ultrapure water (E2-20 TJ, EPED, Taiwan, China) for one hour and was filtered through a 0.22-μm PTFE syringe filter (SCAA-213, ANPEL, Shanghai, China) to remove insoluble components and prepare a water-soluble PM_2.5_ extract. Blank filters were extracted using the same procedure.

### 2.3. Chemical Analysis

A total of 2.27 cm^2^ of each filter was punched with a special perforator, organic carbon (OC) and elemental carbon (EC) were analyzed using a carbon analyzer (RT-4, Sunset Laboratory, Portland, OR, USA).

Two cm^2^ of each filter was added into a microwave digestion tube with 5 mL of 65% nitric acid and digested at 190 °C for 25 min. The digestion solution was then vacuum filtrated (PTFE, 0.22 μm) three times after cooling to room temperature and diluted to 45 mL. The contents of heavy metals and transition metals (Al, Zn, V, Cr, Mn, Co, Ni, Cu, As, Se, Cd, Ba, and Pb) in the samples were analyzed by inductively coupled plasma mass spectrometry (ICP-MS, Thermo Fisher, Waltham, MA, USA, X2 series).

Water soluble organic carbon (WSOC) analysis was performed using a total organic carbon analyzer (TOC-Lcph/cpn, Shimadzu, Kyoto, Japan) with 20 mL of the extract prepared in 2.1.

### 2.4. Light Absorption and Fluorescence Analysis

The light absorption spectra of the extracts were measured in the range of 200–800 nm using a UV-Vis spectrophotometer. The absorbance coefficient (Abs_λ_) of the extract solution at a given wavelength (λ) was calculated by the following equation:

(1) $${\mathrm{Abs}}_{\lambda }{=\;(A}_{\lambda }{\;-\;A}_{700})\;\times \frac{V_{l}}{V_{a}\cdot l}\times \;\ln10.$$

A_700_ is the reference for the baseline drift of the system, V_l_ is the volume of the sample extracted with ultrapure water, V_a_ is the volume of sampled air, and l is the optical path length (1 cm) of the quartz cuvette. The absorbance at 365 nm was chosen to calculate Abs_365_, which was used to evaluate the absorption of water-soluble aromatic substances [25]. The absorption Ångström exponent (AAE) can be used to describe the wavelength dependence of the absorption and is calculated as follows:

(2) $${\mathrm{Abs}}_{\lambda \;}=\;K\cdot \lambda^{-\mathrm{AAE}}.$$

K is the scale constant, and λ ranges from 300–500 nm. The value of Abs_λ_ depends on the size and composition of the particles.

The mass absorption efficiency (MAE_λ_, m^2^ g^−1^) of aromatic substances at 365 nm was calculated using the following equation:

(3) $${\text{MAE}}_{\lambda }\;=\;\frac{{\text{Abs}}_{\lambda }}{\text{WSOC}}.$$

The excitation-emission matrix (EEM) of the extracts was characterized using a fluorescence spectrophotometer (Cary Eclipse, Agilent, Santa Clara, CA, USA) with an excitation (Ex) wavelength range of 235–500 nm and an emission (Em) wavelength range of 300–600 nm. Scanning Ex and Em wavelengths were scanned in wavelength increments of 5 and 2 nm, respectively. The slit width was 5 nm, the scan speed was 9600 nm min^−1^, and the photomultiplier tube (PMT) detector voltage was 600 V. System bias was calibrated by subtracting the Riley and Raman scattering peaks and the blank sample (internal filter correction). Fluorescence intensity (Ff, RU: Raman unit, m^2^ g^−1^) was calculated using parallel factor (PARAFAC) model, and the resulting EEM data were processed using the drEEM toolbox (version 0.1.0, MATLAB R2014b) [26]. The PARAFAC model is performed under non-negativity constraints and can be used to decompose the EEM fluorescence signal into independent chemical components, based on the theory of trilinear decomposition, a mathematical model implemented using an alternating least-squares algorithm. The solution of PARAFAC analysis is unique and useful in the study of unknown complex systems. The parallel factorial model (sample × excitation wavelength × emission wavelength, 131 × 54 × 169) in this study was tested by residual analysis, visualization of spectral loadings, and core coherence analysis to obtain three independent fluorescence components; a halves analysis showed that this model was stable.

### 2.5. OP of PM_2.5_

The specific steps of the DTT method used in this study were as follows. First, 1 mL of the PM_2.5_ extract sample from 2.1 and 3.5 mL of phosphate-buffered saline (PBS, 0.1 mmol L^−1^) and 0.5 mL of DTT solution (1 mmol L^−1^) were added to a 10 mL centrifuge tube and heated in a water bath at 37 °C (simulating the human environment). To ensure a linear relationship between the amount of DTT degradation and the reaction time, the reaction is usually performed for 60 min. At 0, 15, 30, 45, and 60 min, 1 mL of the mixture was added to a 5 mL tube with 0.5 mL of trichloroacetic acid solution (TCA, 10%, as a termination agent); 50 μL of 5,5′-dithiobis-2-nitrobenzoic acid (DTNB, as a chromogenic agent) and 2 mL of Tris-HCL buffer (0.4 mol L^−1^, with 20 mM EDTA) were then added immediately with shaking and the reaction was protected from light for 5 min. The solution was added to a 96-well plate, and the absorbance was measured at 412 nm in a multifunctional microplate reader (SpectraMax iD3, Molecular Devices, San Jose, CA, USA). Both samples and the blank control were conducted made in 6 parallels. The DTT degradation rate of each sample can be calculated from the absorbance. Standard curves were prepared for each group of experiments to ensure that the correlation coefficients of the standard curves for each group of experiments were above 0.99.

The rate of DTT consumption was mass-standardized to DTT_m_ (unit: nmol min^−1^ μg^−1^) to characterize the OP per unit mass of PM_2.5_, which is analogous to the “density” of the OP. The rate of DTT consumption was volumetrically standardized to obtain DTT_v_ (unit: nmol min^−1^ m^−3^), which is considered to be directly related to the health effects of human exposure [27]. DTT_m_ and DTT_v_ were used to characterize the OP of PM_2.5_. They can be calculated by the following equations [28]:

(4) $${\mathrm{DTT}}_{m}=\frac{r_{s}{\;-\;r}_{b}}{M\;\times \;\frac{A_{s}}{A_{t}}\;\times \;\frac{V_{r}}{V_{e}}},$$

(5) $${\mathrm{DTT}}_{v}=\frac{r_{s}{\;-\;r}_{b}}{V_{a}\;\times \;\frac{A_{s}}{A_{t}}\;\times \;\frac{V_{r}}{V_{e}}},$$

where r_s_ and r_b_ are the DTT consumption rates of the sample and blank, respectively; V_a_ and M are the total sampling air volume and the total particle mass, respectively; A_s_ and A_t_ are the areas of the sample and total filter, respectively; V_r_ and V_e_ are the sample volume participating in the reaction and extraction volume, respectively.

### 2.6. Statistical Analysis

The Pearson correlation analysis, analysis of variance (ANOVA), and the multiple linear regression model were performed in the corresponding statistical analyses. A *p* value of <0.05 was considered as the significance level. All the statistics were implemented using the Statistical Program for Social Sciences (SPSS, Version 25, IBM, Armonk, NY, USA).

## 3. Results and Discussion

### 3.1. PM_2.5_ Mass Concentration and Chemical Component

The study site was sampled from March to December 2016. Spring was considered to run from March to May, summer from June to August, autumn from September to October, and winter from November to December. November was considered a part of winter because the meteorological data revealed that the average monthly temperature was 9.9 °C in November 2016, which was closer to the average monthly temperature in December 2016 (7.6 °C) than in October 2016 (18.5 °C).

In 2016, the annual average of the PM_2.5_ mass concentration in Nanjing was 73 μg m^−3^, the seasonal averages of the PM_2.5_ mass concentration were 107 μg m^−3^ in spring, 55 μg m^−3^ in summer, 46 μg m^−3^ in autumn, and 55 μg m^−3^ in winter. The lowest PM_2.5_ mass concentration was presented in autumn and the highest one was presented in spring, which was twice that of the other seasons.

Several transition and heavy metals were measured, including: Al, Zn, V, Cr, Mn, Co, Ni, Cu, As, Se, Cd, Ba, and Pb. The concentrations of several metals were low, namely V, As, and Se. Seasonal differences in the concentration of Cu were not significant, and the lowest Cu concentration was observed in summer (0.18 μg m^−3^). The concentration of Mn was highest in spring, followed by autumn, summer, and winter (Table A1).

**Figure 1:**
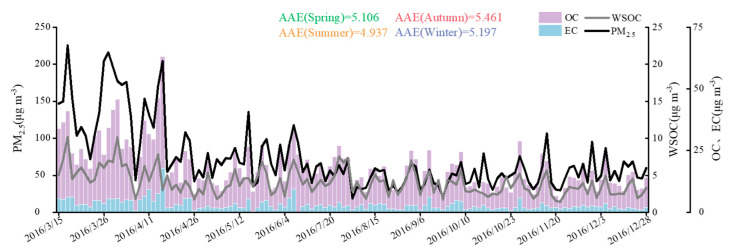
The concentrations of organic carbon (OC), elemental carbon (EC), water soluble organic carbon (WSOC) and ambient fine particle matter (PM_2.5_) in Nanjing from March to December 2016.

The patterns of variation in OC and WSOC concentrations were similar (Figure 1). The annual average concentration of EC was 3.03 μg m^−3^ and that for OC was 15.26 μg m^−3^. The annual average concentration of WSOC was 4.07 μg m^−3^, which accounted for 27% of OC and was similar to the value obtained in 2017 (5.5 μg m^−3^) [29]. WSOC concentrations were highly correlated with PM_2.5_ mass concentrations, especially in winter and spring. The highest WSOC concentration (4.74 μg m^−3^) was in spring, and the lowest (3.15 μg m^−3^) was in winter, WSOC concentrations in summer (4.34 μg m^−3^) and autumn (3.45 μg m^−3^) were in between.

The light absorbing organic carbon in WSOC has adverse health risks, which, according to previous studies, was a multi-component mixture [30,31]. It has some complex component, such as HULIS, nitroaromatic compounds, and aromatic compounds [32]. Benzene structures with multiple conjugation systems have significant light absorption at UV 365 nm [33]. The absorbance efficiency of these water-soluble chromophores with aromatic structures can be estimated by the absorbance at 365 nm, with an annual average Abs_365_ of 6.06 Mm^−1^. Abs_365_ showed the following rank order among seasons: spring (7.27 Mm^−1^) > winter (5.75 Mm^−1^) > summer (5.63 Mm^−1^) > autumn (4.40 Mm^−1^). We speculate that levels of water-soluble UV absorbing aromatic structures are highest in spring because of the dry, dusty weather, and lowest in summer because of the abundant precipitation. The year-round pattern in WSOC was nearly identical to that for PM_2.5_ concentrations and OC (Figure 1). The value of AAE can reflect the wavelength dependence of extracts, with an annual average AAE of 5.175; the highest value of AAE was in autumn (AAE = 5.461), and the lowest was in summer (AAE = 4.937), indicating that the wavelength dependence of extracts was lower in summer compared with autumn and winter. The value of AAE is similar to that reported in Gwangju (AAE = 5.3) [34] but lower than that for Beijing (AAE = 7.28) [33].

**Figure 2:**
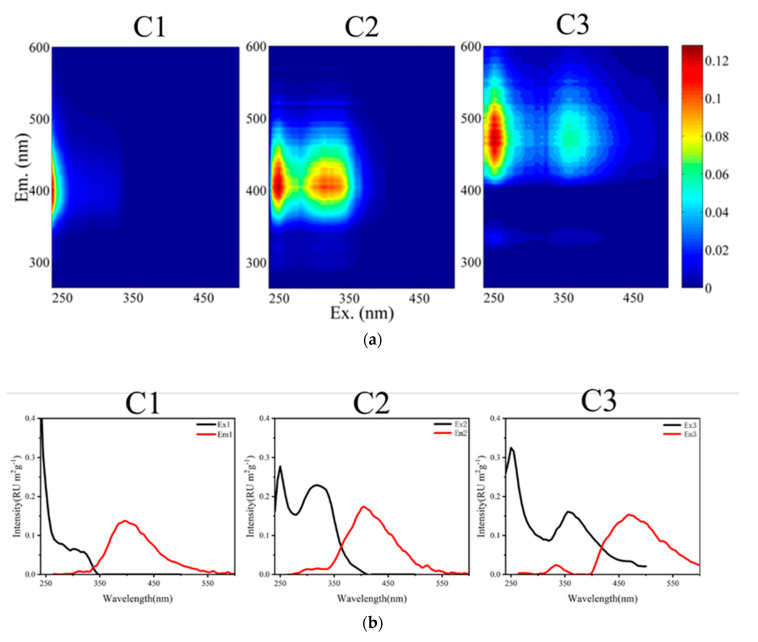
(**a**) Three fluorescent components (C1, C2, and C3) identified by the EEM; (**b**) The relationship between the fluorescence intensity and wavelength of the excitation (Ex) and emission (Em) wavelengths of each fluorescent component.

**Figure 3:**
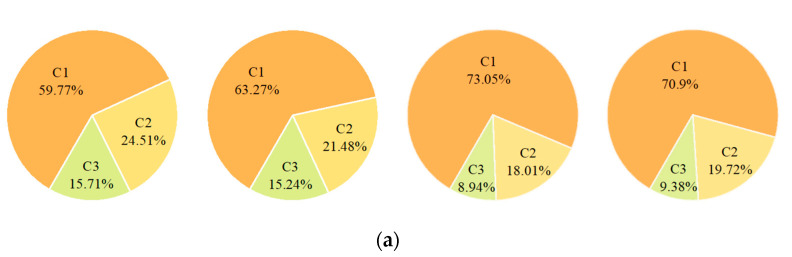

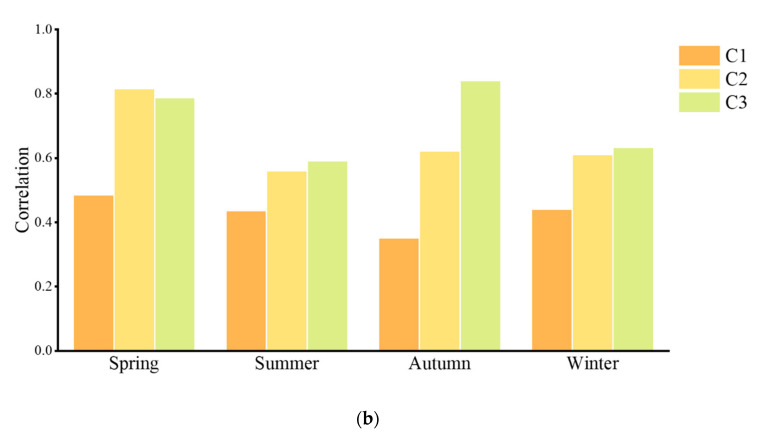
(**a**) Proportion of three fluorescent component (C1, C2, and C3) in the four seasons; (**b**) Pearson correlation coefficients of C1, C2, and C3 with DTT_v_ in four seasons, respectively.

HULIS are organic substances in PM_2.5_ with high OP. Although HULIS species share similar chemical properties, they vary in their ability to promote ROS production. The EEM method is capable of detecting the optical and structural properties of chromophores in complex organics [35]. To analyze the effect of HULIS with different light absorption and fluorescence properties in WSOC on the OP, we used the EEM method and the PARAFAC model, and three fluorescent components were identified by semi-validation. The fluorescence components of the PM_2.5_ samples from Nanjing in 2016 were thus divided into three categories. The contour maps of the three characteristic fluorescent components are shown in Figure 2. In Figure 2a, the first and second components are HULIS chromophores, and the third component has both HULIS and protein-like chromophores (denoted C1, C2, and C3, respectively); the C2 spectra were highly similar to typical HULIS spectra and were also consistent with previous studies [35,36]. In Figure 2b, the maximum Ex and Em wavelengths of the three fluorescence components are shown. The peak in the C1 fluorescence spectrum is located at Ex/Em = 235/395, which is similar to the results of other studies (Ex/Em ≤ 250/400) with spectra of microbial or anthropogenic humic-like substances [37]. This peak has also been shown to correspond to a quinone-like component [38]. C2 has two typical peaks—Ex/Em = 250/405 and Ex/Em = 310/405—which are likely related to the decomposition of photodegradation from macromolecules [39] and might thus correspond to a terrestrial humic-like component [37,40]. The two peaks of C3 have larger Ex and Em wavelengths, indicating that C3 corresponds to a more unsaturated substance containing more unsaturated functional groups and conjugate systems than C1 and C2. Ex/Em = 250/335 corresponds to a protein-like tryptophan [41], and Ex/Em = 365/465 corresponds to a terrestrial humic or fulvic acid-like component. Previous studies have confirmed biomass burning, residual oil combustion, and secondary formation are dominant sources of humic-like component [42,43]. There were seasonal differences in the fluorescence emitting components of PM_2.5_ in Nanjing (Figure 3a). C1 was the highest in the four seasons (Spring = 59.77%, Summer = 63.27%, Autumn = 73.05%, Winter = 70.9%), and C3 was the lowest (Spring = 15.71%, Summer = 15.24%, Autumn = 8.94%, Winter = 9.38%). The proportion of C1 was highest in autumn, indicating that the proportion of microbial or anthropogenic humic-like substances in PM_2.5_ in autumn was higher than in the other seasons. The highest proportion of humic-like substances from terrestrial contemporary carbon sources (i.e., biomass burning and biogenic emission) was also observed in autumn in Seoul [44]. The proportions of C2 and C3 were highest in summer. Similarly, terrestrial humic-like substances and protein-like tryptophan accounted for a higher proportion of the PM_2.5_ fluorescent chromogenic component in summer than in other seasons.

### 3.2. OP of PM_2.5_

The OP of PM_2.5_ in this study was assessed by the DTT method, using volume-standardized and mass-standardized DTT degradation rates (DTT_m_ and DTT_v_, respectively), which are proportional to ROS production. In 2016, the average annual DTT_m_ in Nanjing (*n* = 121) was 0.02 nmol min^−1^ μg^−^^1^ (median: 0.02, range: 0.01–0.06 nmol min^−1^ μg^−1^). The average annual DTT_v_ was 1.16 nmol min^−1^ m^−3^ (median: 1.15, range: 0.52–2.09 nmol min^−1^ m^−3^), and the PM_2.5_ concentration ranged from 18–226 μg m^−3^. These values were higher than those in cities in developed countries, such as Atlanta (DTT_v_ = 0.31 nmol min^−1^ m^−3^, DTT_m_ = 0.01–0.05 nmol min^−1^ μg^−1^) where the PM_2.5_ concentration ranges from 5–25 μg m^−3^ [16,45], Los Angeles (DTT_v_ = 0.13 nmol min^−1^ m^−3^, DTT_m_ = 0.014–0.024 nmol min^−1^ μg^−1^) where the PM_2.5_ concentration ranges from 5–10 μg m^−3^ [46]; Lecce in southeastern Italy (DTT_v_ = 0.40 nmol min^−1^ m^−3^, DTT_m_ = 0.015 nmol min^−1^ μg^−1^) where the PM_2.5_ concentration ranges from 18–42 μg m^−3^ [47], and Europe (DTT_v_ = 0.06–0.69 nmol min^−1^ m^−3^) [48]. However, values in Nanjing were lower than other cities, such as Patiala (DTT_v_ = 1.3–7.2 nmol min^−1^ m^−3^, DTT_m_ = 0.013–0.050 nmol min^−1^ μg^−1^) where the PM_2.5_ concentration ranges from 59–312 μg m^−3^ [49] and Bangkok (DTT_v_ = 1.25–3.82 nmol min^−1^ m^−3^, DTT_m_ = 0.018–0.119 nmol min^−1^ μg^−1^). The particle matter in developed countries has lower OP than that measured in Nanjing, but compared with other developing countries such as India and Thailand, the OP is lower in Nanjing.

**Figure 4:**
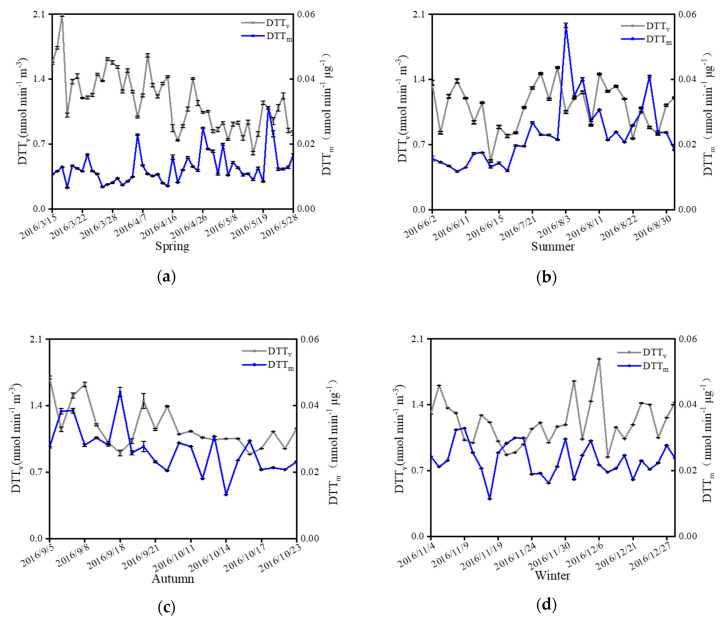
The DTT_v_ and DTT_m_ in Nanjing in spring (**a**), summer (**b**), autumn (**c**), and winter (**d**).

The measured OP in Nanjing is lower than that of the Bohai Sea in Northern China (DTT_v_ = 0.16–14.47 nmol min^−1^ m^−^^3^, DTT_m_ = 0.002–0.084 nmol min^−1^ μg^−1^) where the PM_2.5_ concentration ranges from 26–202 μg m^−3^ [50]. Although the measured OP in Nanjing is lower than Beijing in 2017 (DTT_v_ = 12.26 nmol min^−1^ m^−3^, DTT_m_ = 0.13 nmol min^−1^ μg^−1^) when the PM_2.5_ concentration was 113.8 ± 62.7 μg m^−3^ [51], it was higher than Beijing in 2014 (DTT_v_ = 0.11–0.49 nmol min^−1^ m^−3^) when the PM_2.5_ concentration ranged from 50–410 μg m^−3^ [52]. The measured OP was higher in Nanjing than the one in Shanghai, which is also in the YRD (DTT_v_ = 0.19 nmol min^−1^ m^−3^), but was lower than the OP of the North China Plain, which presumably stems from the increased use of winter heating and frequency of motor vehicle cold starts in the North China Plain. The DTT_v_ in Nanjing shows the following rank order: winter (average: 1.21, median: 1.19, range: 0.84–1.89 nmol min^−1^ m^3^) > spring (average: 1.17, median: 1.15, range: 0.60–2.09 nmol min^−1^m^−3^) > autumn (average: 1.16, median: 1.11, range: 0.89–1.70 nmol min^−1^m^−3^) > summer (average: 1.11, median: 1.17, range: 0.52–1.53 nmol min^−1^ m^−3^) (Table A1). DTT_v_ was higher in spring and winter than in summer and autumn (Figure 4); by contrast, DTT_m_ was highest in summer and lowest in spring. Similarly, Paraskevopoulou et al. found that DTT_v_ was lowest in summer (0.24 ± 0.10 nmol min^−1^ m^−3^) and autumn (0.24 ± 0.11 nmol min^−1^ m^−3^) in Athens [23]. In Los Angeles and Atlanta, DTT_v_ was higher in cold seasons than in warm seasons [45,46].

A positive correlation between DTT_v_ and PM_2.5_ mass concentrations was observed, and the strength of the positive correlation varied among seasons. The lower temperatures, higher humidity, and slower wind speeds in spring and winter are not conducive to the diffusion of pollutants. Consequently, the rate of DTT degradation per unit volume is greater because of the higher concentration of aerosol particles. In contrast, the rate of DTT degradation in summer and autumn is lower. DTT_m_ is negatively correlated with PM_2.5_ mass concentration, which is a opposite pattern compared to DTT_v_.

### 3.3. Associations between PM_2.5_ Components and OP

**Figure 5:**
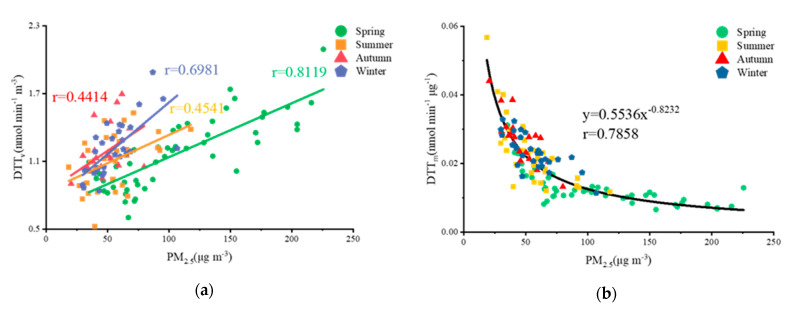
(**a**) Correlation between DTT_v_ and PM_2.5_ mass concentration over four seasons in Nanjing 2016; (**b**) Correlation between DTT_m_ and PM_2.5_ mass concentration over the entire year.

DTT_v_ is positively correlated with the PM_2.5_ mass concentration. The correlation between DTT_v_ and PM_2.5_ mass concentration was the highest in spring, with Pearson’s correlation coefficient above 0.8, 0.7 in winter, and only around 0.4 in summer and autumn (Figure 5a). The OP and adverse health effects caused by PM_2.5_ cannot be evaluated exclusively by the mass concentration, as differences in OP may stem from differences in the redox activity of each compound [53]. Unlike DTT_v_, DTT_m_ decreases as the PM_2.5_ mass concentration increase, and a clear negative non-linear correlation provided the best fit (Figure 5b), which is similar to the power function between DTT_m_ and total suspended particulate mass concentration observed by Jiaqi et al. [54]. Below, the relationships between specific components in PM2.5 and OP among seasons are discussed, and the relative contributions of these components are evaluated.

**Figure 6:**
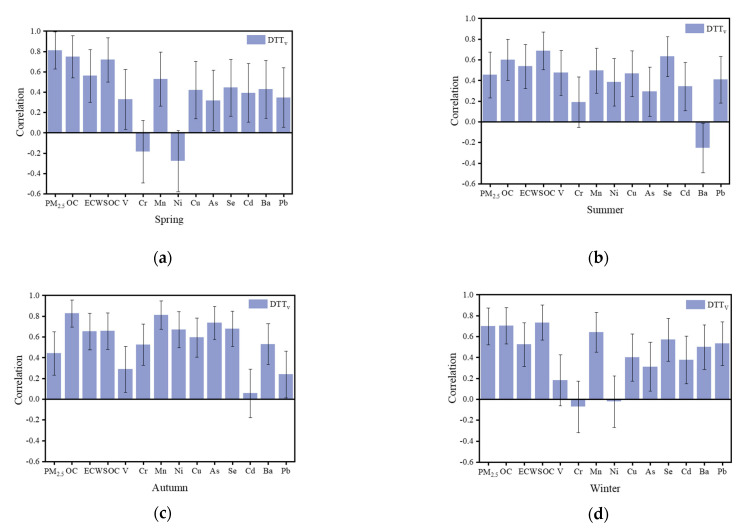
Correlations between DTT_v_ with OC, EC, WSOC, metals, and PM_2.5_ mass concentrations in spring (**a**), summer (**b**), autumn (**c**), and winter (**d**).

The correlations of some metals, including V, Mn, Ni, Cu, As, Se, Cd, Ba, and Pb as well as OC, EC, and WSOC with DTT_v_ are shown in Figure 6. In spring, DTT_v_ had a correlation of 0.81 with PM_2.5_ mass concentration and correlations of 0.75, 0.56, and 0.72 with OC, EC, and WSOC, respectively; the correlations with each metal were all around 0.4. In summer, the correlation between DTT_v_ and PM_2.5_ mass concentration was only 0.45, but the correlation of DTT_v_ with OC and WSOC exceeded 0.6, indicating that organic substances in particles contribute more to the OP in summer. In autumn, the correlation of DTT_v_ with PM_2.5_ mass concentration remained low, but the correlation with OC was 0.83; the correlations between metals with DTT_v_ were high, especially with Mn (r = 0.81) and As (r = 0.74). The highest correlations between DTT_v_ with WSOC (r = 0.73) and with Mn were observed in winter and autumn, suggesting that organic and metallic inorganics in the winter PM_2.5_ contribute the most to the OP. Fang et al. found a strong correlation between DTT_v_ and Cu and Mn (r = 0.68; r = 0.65–0.75) [16]. Some heavy metals such as Cu and Mn can promote the generation of ROS, indicating that greater concentrations of these metals correspond to, greater adverse health effects. The dominant sources affecting OP were organic substances in spring and summer and metals in autumn and winter; however, the organic species that contributed significantly to OP remained unclear; consequently, the analytical results of absorbance and fluorescence were used to identify and classify these organic species.

**Figure 7:**
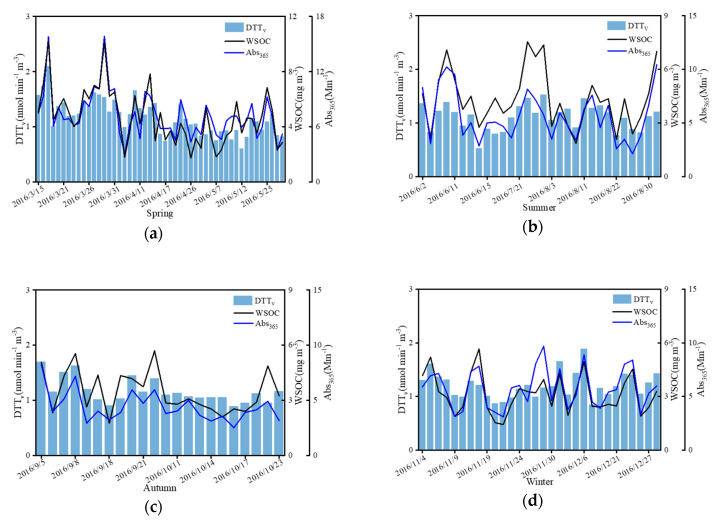
Concentration of WSOC, DTT_v_, and Abs_365_ in spring (**a**), summer (**b**), autumn (**c**), and winter (**d**).

Figure 7 showed the pattern in DTT_v_ and ABS_365_ in the four seasons. Patterns of variation in Abs_365_ and WSOC were similar, and the correlation between Abs_365_ and DTT_v_ was stronger in cooler seasons. The Pearson correlation coefficient between Abs_365_ with DTT_v_ reached 0.83 in autumn, which was the highest among all seasons; this was also much higher than the correlation coefficient between PM_2.5_ mass concentration and DTT_v_ in autumn. For both spring, summer, and winter, the correlation coefficients of Abs_365_ with DTT_v_ exceeded 0.6. This indicates that the water-soluble UV absorbing substances with aromatic structures from source such as biomass burning have the greatest influence on DTT redox activity in autumn, thus explaining the higher OP, just like previous studies [55].

C1, C2, and C3 were identified by the PARAFAC method, which were from different sources and had different contents in PM_2.5_; therefore, their contribution to the OP of PM_2.5_ differed. Although the combined proportion of C2 and C3 was less than that of C1, the correlation between these two components with DTT_v_ was much higher than that of C1 (Figure 3b). The correlation between DTT_v_ with C1 was less than 0.5, but the correlation between DTT_v_ with C2 was 0.82 in spring, which exceeded the correlation between DTT_v_ and PM_2.5_ mass concentration in the same season (r = 0.81). Thus, C2 made the greatest contribution to the OP of PM_2.5_ in spring 2016 in Nanjing; thus, C2 can be used to predict the health effects of PM_2.5_ by measuring terrestrial humic-like substances. Unlike spring, the highest correlations observed in the other three seasons were between C3 components and DTT_v_, especially in autumn when the Pearson correlation coefficient reached 0.84, which was higher than the correlations of DTT_v_ with the PM_2.5_ mass concentration (r = 0.44) and Mn (r = 0.81). This indicates that Mn, water-soluble UV absorbing aromatic structures, humic-like substances, and protein-like tyrosine affect the production of ROS by PM_2.5_ in autumn. The results suggest that these HULIS greatly affected the degradation of DTT, which may stem from their highly conjugated properties and abundant functional groups; as a result, their contribution to OP was not negligible. The role played by these macromolecules of organic matter should also be given increased consideration when estimating the toxicity of atmospheric particulate matter.

In this study, MLR analysis was used between target species of the components (independent variables) and DTT_v_ (dependent variable) for the four seasons to identify substances that primarily drive DTT redox activity, and statistically regression models (*p* < 0.05) were retained. The metallic elements Al, Zn, V, Cr, Mn, Co, Ni, Cu, As, Se, Cd, Ba, and Pb, as well as Abs_365_ and C1-3, were used as independent variables to investigate their specific contribution to the production of DTT redox activity. Table 1 shows the best-performing output models for each season. The standardized coefficients show that aromatic compounds, HULIS, Cu, and Mn were positively correlated with DTT consumption, but metal Cr and V were negatively correlated with DTT consumption (β_Cr_ = −0.17, β_V_ = −0.356). During the spring, each additional unit of terrestrial HULIS resulted in 0.8 units of DTT degradation, but each unit of Cr reduction resulted in 0.2 units of DTT degradation. The complexation of Cr with HULIS might reduce the ability of HULIS to degrade DTT [56], which explains the negative correlation between Cr and DTT_v_. In summer, water-soluble UV absorbing substances with aromatic structures and Cu explained 60% of the DTT redox contribution. Cu is mainly derived from the tire wear of land-based traffic sources [57]. DTT degradation is mainly caused by fulvic acid-like component and Mn in autumn, and they together caused 0.9 units of DTT degradation per unit increase; greater concentrations of V inhibited DTT degradation. Fulvic acid-like component might contains a mass of “total acidic groups”, such as carboxyl groups (COOH) and phenolic hydroxyl groups (PhOH), we assume these components react with V and are thus consumed [58]. Therefore, the concentration of V was negatively correlated with the degradation of DTT at low concentrations (0.02 μg m^−3^) in autumn. V is an indicator of ship emissions that is associated with fine particles [59]. In winter, the redox activity of DTT was mainly affected by the metals Mn and Cu (β_Mn_ = 0.404, β_Cu_ = 0.291), which was different from the pattern observed in spring and summer at higher temperatures. Mn can have complex emission sources. Although Mn is usually considered as an element that originates from the crust, metallurgical emissions may be the largest contributor of Mn to the atmosphere [57,60].

## 4. Conclusions

The annual average PM_2.5_ mass concentration in Nanjing was 73 μg m^−3^, which was higher than that documented for other cities in the YRD; there were seasonal differences in the OP of PM_2.5_ from volume-standardized DTT degradation, and the rank order was as follows: winter > spring > autumn > summer. The correlation between the OP of PM_2.5_ and PM_2.5_ mass concentration was not strong. The contribution of metals, aromatic substances, and humic-like and protein-like substances (C1 > C2 > C3) in OP differed. C2 was found to make the highest OP contribution to PM_2.5_ in spring, while C3 made the highest OP contribution to PM_2.5_ in autumn, the water-soluble UV absorbing aromatic groups had the greatest effect on OP in summer, Mn and Cu contributed the most to OP in winter. These findings indicate that predicting the health risks of PM_2.5_ using the mass concentration of PM_2.5_ is not a robust approach. The substances that dominate the OP of particulate matter vary from season to season. This study is the first to evaluate the OP of humic-like and protein-like substances in PM_2.5_ in Nanjing characterized by EEM using the DTT method. However, our study has several limitations. There was only one sampling site, the method of source apportionment could be improved, and the results are preliminary. Additional research is needed to measure the specific contributions of various substances to OP and to outline appropriate preventive measures.

## Figures and Tables

**Table 1:** Model of multiple linear regression output with DTT_v_ as the dependent variable and organic target components and metals as independent variables.

Season	Component	Standardized Coefficients	*p*-Value	R^2^
Spring	C2	0.812	0.000	0.695
	Cr	−0.170	0.042	
Summer	Abs_365_	0.617	0.000	0.596
	Cu	0.419	0.002	
Autumn	C3	0.555	0.003	0.916
	Abs_365_	0.286	0.047	
	Mn	0.387	0.001	
	V	−0.356	0.001	
Winter	C3	0.399	0.007	0.628
	Cu	0.291	0.024	
	Mn	0.404	0.007	

## Data Availability

This study did not report any data.

## Appendix A

**Table A1:** The WSOC, OC, EC, TC, IC, metals, Abs_365_, MAE_365_, AAE, DTT_v_, DTT_m_, and PM_2.5_ mass concentrations in all seasons.

Constituents	Unit	Spring Mean ± SD (*n* = 49)	Summer Mean ± SD (*n* = 30)	Autumn Mean ± SD (*n* = 22)	Winter Mean ± SD (*n* = 30)	Annual Mean ± SD (*n* = 131)
PM_2.5_	μg m^−3^	107.0 ± 52.1	55.0 ± 21.8	46.2 ± 13.3	55.3 ± 18.2	73.0 ± 44.0
WSOC	μg m^−3^	4.7 ± 1.9	4.3 ± 1.6	3.5 ± 1.1	3.2 ± 1.1	4.1 ± 1.7
OC	μg m^−^^3^	19.6 ± 9.4	14.9 ± 5.5	11.4 ± 4.1	11.4 ± 4.6	15.3 ± 7.8
EC	μg m^−3^	4.0 ± 2.8	2.8 ± 1.6	2.3 ± 1.4	2.2 ± 0.9	3.0 ± 2.2
TC	mg L^−1^	5.8 ± 2.1	5.4 ± 1.8	4.4 ± 1.3	4.0 ± 1.1	5.1 ± 1.9
IC	mg L^−1^	0.6 ± 0.2	0.5 ± 0.1	0.5 ± 0.1	0.5 ± 0.1	0.6 ± 0.1
TN	mg L^−1^	12.2 ± 6.4	7.1 ± 4.6	5.3 ± 2.1	6.7 ± 2.4	8.6 ± 5.5
Al	μg m^−3^	5.3 ± 3.4	3.2 ± 1.5	2.0 ± 0.8	3.6 ± 4.0	3.9 ± 3.2
Zn	μg m^−3^	5.0 ± 2.2	3.4 ± 1.8	3.6 ± 2.0	3.5 ± 1.9	4.1 ± 2.2
V	ng m^−3^	33.8 ± 21.1	23.1 ± 17.4	20.9 ± 12.6	12.5 ± 8.5	24.3 ± 20.0
Cr	ng m^−3^	150.2 ± 68.0	119.2 ± 27.0	135.1 ± 60.5	199.2 ± 110.2	151.8 ± 78.1
Mn	ng m^−3^	292.8 ± 150.0	137.1 ± 53.4	157.0 ± 72.8	124.0 ± 43.7	195.7 ± 126.9
Co	ng m^−3^	4.2 ± 2.1	3.8 ± 5.2	3.3 ± 1.5	2.5 ± 0.6	3.6 ± 3.0
Ni	ng m^−3^	61.8 ± 32.6	58.7 ± 13.8	64.5 ± 20.5	66.2 ± 34.5	62.5 ± 28.1
Cu	ng m^−3^	216.2 ± 91.6	178.3 ± 77.0	187.8 ± 146.6	194.8 ± 158.0	197.9 ± 118.6
As	ng m^−3^	30.6 ± 26.5	18.3 ± 12.2	11.9 ± 5.4	17.5 ± 18.6	21.7 ± 20.8
Se	ng m^−3^	24.8 ± 12.0	17.0 ± 6.9	13.8 ± 6.4	12.7 ± 5.3	18.4 ± 10.2
Cd	ng m^−3^	8.9 ± 5.1	8.2 ± 6.1	5.5 ± 3.5	6.8 ± 5.2	7.7 ± 5.3
Ba	ng m^−3^	93.1 ± 36.5	112.9 ± 217.7	58.4 ± 20.0	57.0 ± 17.8	83.5 ± 110.0
Pb	μg m^−3^	0.9 ± 1.0	0.4 ± 0.7	0.3 ± 0.1	0.3 ± 0.2	0.5 ± 0.8
Abs_365_	Mm^−1^	7.3 ± 2.5	5.6 ± 2.3	4.4 ± 1.4	5.8 ± 1.8	6.1 ± 2.4
MAE_365_	m^2^ mg^−1^	1.6 ± 0.4	1.3 ± 0.3	1.3 ± 0.3	1.9 ± 0.3	1.6 ± 0.4
AAE (300–500 nm)	/	5.1	4.9	5.5	5.2	5.2
DTT_v_	nmol min^−1^ m^3^	1.2 ± 0.3	1.1 ± 0.2	1.2 ± 0.2	1.2 ± 0.2	1.1 ± 0.3
DTT_m_	nmol min^−1^ ng^−1^	12.7 ± 5.0	23.1 ± 9.8	26.8 ± 6.9	23.4 ± 5.0	20.0 ± 8.8

## References

[1] Wang G., Kawamura K., Lee S.C., Ho K.F., Cao J.J. (2006). Molecular, Seasonal, and Spatial Distributions of Organic Aerosols from Fourteen Chinese Cities. Environ. Sci. Technol..

[2] Abrams J.Y., Weber R.J., Klein M., Samat S.E., Chang H.H., Strickland M.J., Verma V., Fang T., Bates J.T., Mulholland J.A. (2017). Associations between Ambient Fine Particulate Oxidative Potential and Cardiorespiratory Emergency Department Visits. Environ. Health Perspect..

[3] Bates J.T., Weber R.J., Abrams J., Verma V., Fang T., Klein M., Strickland M.J., Sarnat S.E., Chang H.H., Mulholland J.A. (2015). Reactive Oxygen Species Generation Linked to Sources of Atmospheric Particulate Matter and Cardiorespiratory Effects. Environ. Sci. Technol..

[4] Yang A., Janssen N.A., Brunekreef B., Cassee F.R., Hoek G., Gehring U. (2016). Children’s respiratory health and oxidative potential of PM_2.5_: The PIAMA birth cohort study. Occup. Environ. Med..

[5] Raaschou-Nielsen O., Andersen Z.J., Beelen R., Samoli E., Stafoggia M., Weinmayr G., Hoffmann B., Fischer P., Nieuwenhuijsen M.J., Brunekreef B. (2013). Air pollution and lung cancer incidence in 17 European cohorts: Prospective analyses from the European Study of Cohorts for Air Pollution Effects (ESCAPE). Lancet Oncol..

[6] Donaldson K., Beswick P.H., Gilmour P.S. (1996). Free radical activity associated with the surface of particles: A unifying factor in determining biological activity?. Toxicol. Lett..

[7] Li N., Sioutas C., Cho A., Schmitz D., Misra C., Sempf J., Wang M., Oberley T., Froines J., Nel A. (2003). Ultrafine Particulate Pollutants Induce Oxidative Stress and Mitochondrial Damage. Environ. Health Perspect..

[8] Bates J.T., Fang T., Verma V., Zeng L., Weber R.J., Tolbert P.E., Abrams J.Y., Sarnat S.E., Klein M., Mulholland J.A. (2019). Review of Acellular Assays of Ambient Particulate Matter Oxidative Potential: Methods and Relationships with Composition, Sources, and Health Effects. Environ. Sci. Technol..

[9] Weichenthal S., Crouse D.L., Pinault L., Godri-Pollitt K., Lavigne E., Evans G., van Donkelaar A., Martin R.V., Burnett R.T. (2016). Oxidative burden of fine particulate air pollution and risk of cause-specific mortality in the Canadian Census Health and Environment Cohort (CanCHEC). Environ. Res..

[10] Styszko K., Samek L., Szramowiat K., Korzeniewska A., Kubisty K., Rakoczy-Lelek R., Kistler M., Giebl A.K. (2017). Oxidative potential of PM_10_ and PM_2.5_ collected at high air pollution site related to chemical composition: Krakow case study. Air Qual. Atmos. Health.

[11] Li X., Kuang X.M., Yan C., Ma S., Paulson S.E., Zhu T., Zhang Y., Zheng M. (2019). Oxidative Potential by PM_2.5_ in the North China Plain: Generation of Hydroxyl Radical. Environ. Sci. Technol..

[12] Rattanavaraha W., Rosen E., Zhang H., Li Q., Pantong K., Kamens R.M. (2011). The reactive oxidant potential of different types of aged atmospheric particles: An outdoor chamber study. Atmos. Environ..

[13] Vreeland H., Weber R., Bergin M., Greenwald R., Golan R., Russell A.G., Verma V., Sarnat J.A. (2017). Oxidative potential of PM_2.5_ during Atlanta rush hour: Measurements of in-vehicle dithiothreitol (DTT) activity. Atmos. Environ..

[14] Cho A.K., Sioutas C., Miguel A.H., Kumagai Y., Schmitz D.A., Singh M., Eiguren-Fernandez A., Froines J.R. (2005). Redox activity of airborne particulate matter at different sites in the Los Angeles Basin. Environ. Res..

[15] Kumagai Y., Koide S., Taguchi K., Endo A., Nakai Y., Yoshikawa T., Shimojo N. (2002). Oxidation of Proximal Protein Sulfhydryls by Phenanthraquinone, a Component of Diesel Exhaust Particles. Chem. Res. Toxicol..

[16] Fang T., Verma V., Bates J.T., Abrams J., Klein M., Strickland M.J., Sarnat S.E., Chang H.H., Mulholland J.A., Tolbert P.E. (2016). Oxidative potential of ambient water-soluble PM_2.5_ in the southeastern United States: Contrasts in sources and health associations between ascorbic acid (AA) and dithiothreitol (DTT) assays. Atmos. Chem. Phys..

[17] Liu Q., Lu Z., Xiong Y., Huang F., Zhou J., Schauer J.J. (2020). Oxidative potential of ambient PM_2.5_ in Wuhan and its comparisons with eight areas of China. Sci. Total Environ..

[18] Charrier J.G., Anastasio C. (2012). On dithiothreitol (DTT) as a measure of oxidative potential for ambient particles: Evidence for the importance of soluble transition metals. Atmos. Chem. Phys..

[19] van Amstel M., de Neve W., de Kraker J., Glasbergen P. (2007). Assessment of the potential of ecolabels to promote agrobiodiversity. AMBIO.

[20] Ma Y., Cheng Y., Qiu X., Cao G., Fang Y., Wang J., Zhu T., Yu J., Hu D. (2018). Sources and oxidative potential of water-soluble humic-like substances (HULISWS) in fine particulate matter (PM_2.5_) in Beijing. Atmos. Chem. Phys..

[21] Lin P., Yu J.Z. (2011). Generation of reactive oxygen species mediated by humic-like substances in atmospheric aerosols. Environ. Sci. Technol..

[22] Lyu Y., Guo H., Cheng T., Li X. (2018). Particle Size Distributions of Oxidative Potential of Lung-Deposited Particles: Assessing Contributions from Quinones and Water-Soluble Metals. Environ. Sci. Technol..

[23] Paraskevopoulou D., Bougiatioti A., Stavroulas I., Fang T., Lianou M., Liakakou E., Gerasopoulos E., Weber R., Nenes A., Mihalopoulos N. (2019). Yearlong variability of oxidative potential of particulate matter in an urban Mediterranean environment. Atmos. Environ..

[24] Daellenbach K.R., Uzu G., Jiang J., Cassagnes L.E., Leni Z., Vlachou A., Stefenelli G., Canonaco F., Weber S., Segers A. (2020). Sources of particulate-matter air pollution and its oxidative potential in Europe. Nature.

[25] Hecobian A., Zhang X., Zheng M., Frank N., Edgerton E.S., Weber R.J. (2010). Water-Soluble Organic Aerosol material and the light-absorption characteristics of aqueous extracts measured over the Southeastern United States. Atmos. Chem. Phys..

[26] Murphy K.R., Stedmon C.A., Graeber D., Bro R. (2013). Fluorescence spectroscopy and multi-way techniques. PARAFAC. Anal. Methods.

[27] Fang T., Zeng L., Gao D., Verma V., Stefaniak A.B., Weber R.J. (2017). Ambient Size Distributions and Lung Deposition of Aerosol Dithiothreitol-Measured Oxidative Potential: Contrast between Soluble and Insoluble Particles. Environ. Sci. Technol..

[28] Wang J., Lin X., Lu L., Wu Y., Zhang H., Lv Q., Liu W., Zhang Y., Zhuang S. (2019). Temporal variation of oxidative potential of water soluble components of ambient PM_2.5_ measured by dithiothreitol (DTT) assay. Sci. Total Environ..

[29] Xie X., Chen Y., Nie D., Liu Y., Liu Y., Lei R., Zhao X., Li H., Ge X. (2020). Light-absorbing and fluorescent properties of atmospheric brown carbon: A case study in Nanjing, China. Chemosphere.

[30] Yan J., Wang X., Gong P., Wang C., Cong Z. (2018). Review of brown carbon aerosols: Recent progress and perspectives. Sci. Total Environ..

[31] Chen Y., Xie X., Shi Z., Li Y., Gai X., Wang J., Li H., Wu Y., Zhao X., Chen M. (2020). Brown carbon in atmospheric fine particles in Yangzhou, China: Light absorption properties and source apportionment. Atmos. Res..

[32] Huang R.J., Yang L., Cao J., Chen Y., Chen Q., Li Y., Duan J., Zhu C., Dai W., Wang K. (2018). Brown Carbon Aerosol in Urban Xi’an, Northwest China: The Composition and Light Absorption Properties. Environ. Sci. Technol..

[33] Cheng Y., He K.-B., Du Z.-Y., Engling G., Liu J.-m., Ma Y.-l., Zheng M., Weber R.J. (2016). The characteristics of brown carbon aerosol during winter in Beijing. Atmos. Environ..

[34] Park S., Yu G.-H., Lee S. (2018). Optical absorption characteristics of brown carbon aerosols during the KORUS-AQ campaign at an urban site. Atmos. Res..

[35] Chen Q., Miyazaki Y., Kawamura K., Matsumoto K., Coburn S., Volkamer R., Iwamoto Y., Kagami S., Deng Y., Ogawa S. (2016). Characterization of Chromophoric Water-Soluble Organic Matter in Urban, Forest, and Marine Aerosols by HR-ToF-AMS Analysis and Excitation-Emission Matrix Spectroscopy. Environ. Sci. Technol..

[36] Matos J.T.V., Freire S.M.S.C., Duarte R.M.B.O., Duarte A.C. (2015). Natural organic matter in urban aerosols: Comparison between water and alkaline soluble components using excitation–emission matrix fluorescence spectroscopy and multiway data analysis. Atmos. Environ..

[37] Stedmon C.A., Markager S. (2005). Resolving the variability in dissolved organic matter fluorescence in a temperate estuary and its catchment using PARAFAC analysis. Limnol. Oceanogr..

[38] Mladenov N., Alados-Arboledas L., Olmo F.J., Lyamani H., Delgado A., Molina A., Reche I. (2011). Applications of optical spectroscopy and stable isotope analyses to organic aerosol source discrimination in an urban area. Atmos. Environ..

[39] Chen Q., Ikemori F., Mochida M. (2016). Light Absorption and Excitation–Emission Fluorescence of Urban Organic Aerosol Components and Their Relationship to Chemical Structure. Environ. Sci. Technol..

[40] Yu H., Liang H., Qu F., Han Z.-s., Shao S., Chang H., Li G. (2015). Impact of dataset diversity on accuracy and sensitivity of parallel factor analysis model of dissolved organic matter fluorescence excitation-emission matrix. Sci. Rep..

[41] Salve P.R., Lohkare H., Gobre T., Bodhe G., Krupadam R.J., Ramteke D.S., Wate S.R. (2011). Characterization of Chromophoric Dissolved Organic Matter (CDOM) in Rainwater Using Fluorescence Spectrophotometry. Bull. Environ. Contam. Toxicol..

[42] Zhou X., Zhang L., Tan J., Zhang K., Mao J., Duan J., Hu J. (2018). Characterization of humic-like substances in PM_2.5_ during biomass burning episodes on Weizhou Island, China. Atmos. Environ..

[43] Tan J., Xiang P., Zhou X., Duan J., Ma Y., He K., Cheng Y., Yu J., Querol X. (2016). Chemical characterization of humic-like substances (HULIS) in PM_2.5_ in Lanzhou, China. Sci. Total Environ..

[44] Yan G., Kim G. (2017). Speciation and Sources of Brown Carbon in Precipitation at Seoul, Korea: Insights from Excitation–Emission Matrix Spectroscopy and Carbon Isotopic Analysis. Environ. Sci. Technol..

[45] Verma V., Fang T., Guo H., King L., Bates J.T., Peltier R.E., Edgerton E., Russell A.G., Weber R.J. (2014). Reactive oxygen species associated with water-soluble PM_2.5_ in the southeastern United States: Spatiotemporal trends and source apportionment. Atmos. Chem. Phys..

[46] Hu S., Polidori A., Arhami M., Shafer M.M., Schauer J.J., Cho A., Sioutas C. (2008). Redox activity and chemical speciation of size fractioned PM in the communities of the Los Angeles-Long Beach harbor. Atmos. Chem. Phys..

[47] Chirizzi D., Cesari D., Guascito M.R., Dinoi A., Giotta L., Donateo A., Contini D. (2017). Influence of Saharan dust outbreaks and carbon content on oxidative potential of water-soluble fractions of PM_2.5_ and PM_10_. Atmos. Environ..

[48] Jedynska A., Hoek G., Wang M., Yang A., Eeftens M., Cyrys J., Keuken M., Ampe C., Beelen R., Cesaroni G. (2017). Spatial variations and development of land use regression models of oxidative potential in ten European study areas. Atmos. Environ..

[49] Patel A., Rastogi N. (2018). Oxidative potential of ambient fine aerosol over a semi-urban site in the Indo-Gangetic Plain. Atmos. Environ..

[50] Liu W., Xu Y., Liu W., Liu Q., Yu S., Liu Y., Wang X., Tao S. (2018). Oxidative potential of ambient PM_2.5_ in the coastal cities of the Bohai Sea, northern China: Seasonal variation and source apportionment. Environ. Pollut..

[51] Yu S., Liu W., Xu Y., Yi K., Zhou M., Tao S., Liu W. (2019). Characteristics and oxidative potential of atmospheric PM_2.5_ in Beijing: Source apportionment and seasonal variation. Sci. Total Environ..

[52] Liu Q., Baumgartner J., Zhang Y., Liu Y., Sun Y., Zhang M. (2014). Oxidative potential and inflammatory impacts of source apportioned ambient air pollution in Beijing. Environ. Sci. Technol..

[53] Wang Y., Wang M., Li S., Sun H., Mu Z., Zhang L., Li Y., Chen Q. (2020). Study on the oxidation potential of the water-soluble components of ambient PM_2.5_ over Xi’an, China: Pollution levels, source apportionment and transport pathways. Environ. Int..

[54] Wang J., Jiang H., Jiang H., Mo Y., Geng X., Li J., Mao S., Bualert S., Ma S., Li J. (2020). Source apportionment of water-soluble oxidative potential in ambient total suspended particulate from Bangkok: Biomass burning versus fossil fuel combustion. Atmos. Environ..

[55] Yan C., Ma S., He Q., Ding X., Cheng Y., Cui M., Wang X., Zheng M. (2021). Identification of PM_2.5_ sources contributing to both Brown carbon and reactive oxygen species generation in winter in Beijing, China. Atmos. Environ..

[56] Lin M., Yu J.Z. (2019). Effect of metal-organic interactions on the oxidative potential of mixtures of atmospheric humic-like substances and copper/manganese as investigated by the dithiothreitol assay. Sci. Total Environ..

[57] Lü S., Zhang R., Yao Z., Yi F., Ren J., Wu M., Feng M., Wang Q. (2012). Size distribution of chemical elements and their source apportionment in ambient coarse, fine, and ultrafine particles in Shanghai urban summer atmosphere. J. Environ. Sci..

[58] Lu X., Johnson W.D., Hook J. (1998). Reaction of Vanadate with Aquatic Humic Substances: An ESR and ^51^V NMR Study. Environ. Sci. Technol..

[59] Zhao M., Zhang Y., Ma W., Fu Q., Yang X., Li C., Zhou B., Yu Q., Chen L. (2013). Characteristics and ship traffic source identification of air pollutants in China’s largest port. Atmos. Environ..

[60] Yue W., Li X., Liu J., Li Y., Zhang G., Li Y. (2008). Source tracing of chromium-, manganese-, nickel- and zinc-containing particles (PM_10_) by micro-PIXE spectrum. J. Radioanal. Nucl. Chem..

